# STRESSORS & COPING STRATEGIES OF ADMINISTRATORS IN CHINESE FAITH-BASED DEMENTIA CARE FACILITIES

**DOI:** 10.1093/geroni/igad104.3725

**Published:** 2023-12-21

**Authors:** Lin Jiang, David Hodge, Robin Bonifas, Fei Sun

**Affiliations:** The University of Texas Rio Grande Valley, Edinburg, Texas, United States; Arizona State University, Phoenix, Arizona, United States; Indiana State University, Terre Haute, Indiana, United States; Michigan State University, East Lansing, Michigan, United States

## Abstract

**Objectives:**

Identifying and meeting the spiritual needs of older adults is a central component of holistic service provision. China has more older adults than any other nation, yet a paucity of research exists on the spiritual needs of Chinese older adults. The purpose of this study was to identify: 1) common spiritual needs among Chinese nursing home residents, including the needs of residents with dementia, 2) the process staff use to identify these needs when residents are unable to verbalize them, and 3) the strategies staff use implement to meet the identified needs.

**Methods:**

To perform this qualitative study, semi-structured interviews were conducted with 21 administrators of faith-based nursing homes, representing facilities that may be disproportionately likely to have residents with dementia. The nursing homes spanned 14 Chinese provinces. Interviews were conducted in the study participants’ native language, translated into English, and analyzed using a constant comparative methodology to identify themes.

**Results:**

Analysis produced five interrelated themes regarding older adults’ spiritual needs, which included the need to: express their faith, receive love and care, have contact with their children, interact with others, and participate in activities. Regarding For residents with dementia who are unable to verbalize their concerns, staff used two strategies to identify spiritual needs: careful observation, and communication with family members. To address the identified needs, staff drew from local resources and attempted to personalize services whenever possible.

**Discussion:**

The findings offer guidance to individuals providing holistic care that addresses Chinese older adults’ spiritual needs.

